# Extracellular vesicles in neurodegenerative diseases: A systematic review

**DOI:** 10.3389/fnmol.2022.1061076

**Published:** 2022-11-24

**Authors:** Alok Raghav, Manish Singh, Goo-Bo Jeong, Richa Giri, Saurabh Agarwal, Sanjay Kala, Kirti Amresh Gautam

**Affiliations:** ^1^Multidisciplinary Research Unit, Department of Health Research, Ministry of Health and Family Welfare, Ganesh Shankar Vidyarthi Memorial Medical College, Kanpur, Uttar Pradesh, India; ^2^Department of Neurosurgery, Ganesh Shankar Vidyarthi Memorial Medical College, Kanpur, Uttar Pradesh, India; ^3^Department of Anatomy and Cell Biology, College of Medicine, Gachon University, Incheon, South Korea; ^4^KPS PG Institute of Medicine, Ganesh Shankar Vidyarthi Memorial Medical College, Kanpur, Uttar Pradesh, India; ^5^Department of Surgery, Ganesh Shankar Vidyarthi Memorial Medical College, Kanpur, Uttar Pradesh, India; ^6^Department of Basic and Applied Sciences, School of Engineering and Sciences, GD Goenka University, Gurugram, Haryana, India

**Keywords:** extracellular vesicles, neurodegenerative disease, therapeutics, biomarker, neurological disease

## Abstract

**Introduction:**

Extracellular vesicles (EVs) are known to have a significant role in the central nervous system (CNS) and neurodegenerative disease.

**Methods:**

PubMed, Scopus, ISI Web of Science, EMBASE, and Google Scholar were used to identify published articles about EV modifications (2012 to Feb 2022).

**Results:**

In total, 1,435 published papers were identified among the searched articles, with 1,128 non-duplicate publications being identified. Following the screening of titles and abstracts, 214 publications were excluded; following the full-text screening of 93 published articles, another 33 publications were excluded. The remaining 60 studies were considered. The kappa statistic of 0.868 indicated that the raters were highly reliable. Furthermore, the inter-reliability and intra-reliability coefficients were found to be 0.931 and 0.908, respectively, indicating strong reliability and consistency between the eligible studies identified by the raters. A total of 27 relevant studies demonstrated the role of EVs as therapeutic and diagnostic biomarkers in neurodegenerative diseases. Of note, 19 and 14 studies, respectively, found EVs to be pioneering in diagnostic and therapeutic roles.

**Discussion:**

EVs play an important role in the central nervous system (CNS), aiding in cell-to-cell communication and serving as a diagnostic marker and therapeutic target in a variety of neurodegenerative diseases. EVs are the home of several proteins [including-synuclein (-syn) and tau proteins], lipids, and genetic materials such as DNA and RNA. The presence of novel miRNAs in EVs suggests biomarkers for the diagnosis and screening of neurodegenerative disorders. Furthermore, EVs play an important role in the pathogenesis of such disorders. This systematic review discussed the current state of EVs’ role in neurological diseases, as well as some preclinical studies on the therapeutic and diagnostic potential of EVs.

## Introduction

**FIGURE 1:**
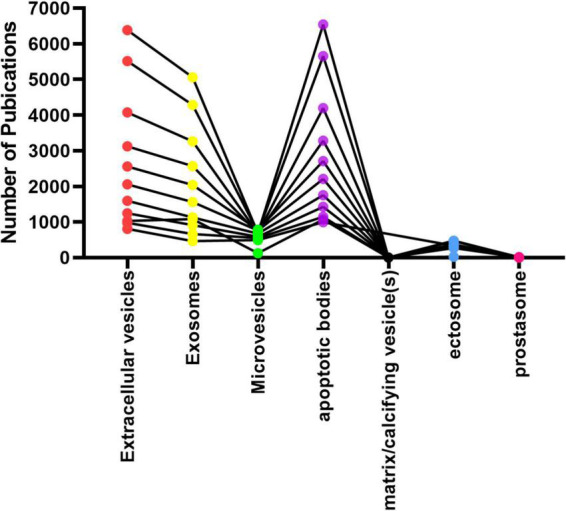
Number of publications for the last 10 years (2012 to 2022) extracted from PubMed.

Extracellular vesicles (EVs) are defined as naturally releasing lipid bilayer delimited vesicles from a cell that cannot replicate. It, which is secreted by the cells, mediates conserved intercellular communications through the content present in their lipid bilayer (Doyle and Wang, 2019). The ISEV nomenclature consensus suggestion is to use “extracellular vesicle” as the “generic term for molecules intuitively launched from the cell that is delimited by a lipid bilayer and cannot replicate” and to modify “EV” based on explicit, specific attributes such as the cell of origin, molecular markers, size, density, function (Théry et al., 2018). EVs secreted as exosomes, microvesicles, and apoptotic bodies from cells exhibit diversity in size, function, secretion pathways, and indigenous cargo (Raghav et al., 2021b). For instance, plasma membrane-derived vesicles are termed microvesicles (150–1,000 nm), while endosome (lumen of internal compartments)-derived vesicles are termed exosomes (< 100 nm) (Théry et al., 2018). Most of the cells release EVs in the form of exosomes rich in biological information including microRNAs (miRNAs), proteins (tetraspanin, membrane-bound, and soluble secreted proteins), peptides, growth factors (GFs), nucleic acids along with other coding information such as lipids, small non-coding RNAs, and long non-coding RNAs that can be exploited for therapeutics and diagnosis purposes (Lötvall et al., 2014; Raghav et al., 2021a). Exosomes are secreted EVs with a cup-shaped spherical morphology and electron microscopic sizes ranging from 30 to 100 nm (Raghav et al., 2021a; Figure 1). These are secreted by a variety of cell types, including hematopoietic cells, primary cells, and cancer cells, as well as biological fluids such as saliva, synovial fluid (SF), serum, plasma, bronchoalveolar lavage (BAL) fluid, urine, amniotic fluid (AF), pleural effusions (PE), and menstrual fluid.

The biogenesis of EVs is a continuous mechanism that starts with inward invagination of the plasma membrane giving rise to early and late endosome bodies by the cells into the cytoplasm (Raghav and Jeong, 2021). Later endosomes further undergo invagination, thereby initiating the formation of membrane-bound intraluminal vesicles (ILVs) within the multivesicular bodies (MVBs) (Raghav and Jeong, 2021). These MVBs combine with the plasma membrane of the cell to release EVs within the extracellular space through exocytosis. The cargo inside the EVs exhibits sorting *via* the endosomal sorting complex required for transport (ESCRT) dependent and independent pathway. The ESCRT-dependent sorting of cargo involves sequential interaction of ESCRT subunits I, II, and III followed by Vps4 protein for initiating the budding and detachment process from the ESCRT III complex (Raghav and Jeong, 2021). Moreover, ESCRT independent sorting mechanism is mediated by tetraspanins, ceramides, and other protein/lipid interplay (Raghav and Jeong, 2021).

Extracellular vesicles were shown to exhibit numerous functions such as deciphering biological pathways involved in inflammation, angiogenesis, programmed cell death, and morphogen transportation (Hill, 2019). This was evident from previously published studies over a decade that a significant amount of research was performed on exploiting EVs for cancer-related studies (Hill, 2019). The EVs have the ability for modification and transportation of desired cargo to desired cells due to their property of flexible lipid bilayer structure, thereby these EVs played important role in the pathogenesis, therapeutics, and diagnosis of several neurological conditions, especially neurodegenerative diseases such as Parkinson’s disease, Alzheimer’s disease, Creutzfeldt–Jakob disease, and amyotrophic lateral sclerosis associated with the misfolded proteins. Besides contributing to the pathogenesis of neurodegenerative diseases, EVs also provide a useful source of biomarkers for such neural complications. A previously published study showed that glioblastoma-derived EVs containing mRNAs/miRNAs at the periphery location can be used as a biomarker for a number of neurological disorders (Redzic et al., 2014).

In Alzheimer’s disease, neural cell-derived EVs are responsible to transport amyloid-ß protein and tau proteins to other cells (DeLeo and Ikezu, 2018). Asai et al. (2015) proved that microglia-derived exosomes contribute to the spreading of tau. In one of the preclinical studies performed on mice having tau aggregation, it was observed that brain-derived exosomes showed the presence of tau *in vivo* that can be further transferred to neurons *ex vivo* through these exosomes (Asai et al., 2015). In another AD-related study, injection of exosome biosynthesis inhibitors showed a significant reduction of Aβ plaque loads in the brain tissues (Dinkins et al., 2014). Several preclinical/clinical studies demonstrated that immunization treatment against EVs containing proteins associated with neurodegenerative diseases could attenuate neurodegenerative pathologies (Valera and Masliah, 2013; van Dyck, 2018; Wang et al., 2019).

The role of EVs from different origins was evaluated in neurodegenerative disease and neurological injury models by several authors in their previously published studies (An et al., 2013; Xin et al., 2013; Zhang et al., 2015). In such models, authors endorsed the neuroprotective behavior of EVs as these lower the episodes of neuropathology and along with also contribute to ameliorating behavioral and cognitive motor deficits (Xin et al., 2013; Doeppner et al., 2015; Zhang et al., 2015). Other animal studies also conferred improvement in cognitive motor outcomes along with showing protective anti-inflammatory phenotype on intravenous (IV) infusion with mesenchymal stem cell (MSC)-derived EVs that are mediated through reduced inflammatory cytokine levels and apoptotic markers (Kim et al., 2016; Ni et al., 2019). Another study also reported that repeated IV doses of endothelial cell-derived EVs showed improved cognitive motor function through the reduction of Aβ deposits (Pan et al., 2020). A similar study also showed that intracerebroventricular administration of EVs derived from neural stem cells enhanced the cognitive-motor function in an animal model of Alzheimer’s disease (Li et al., 2020). The author of a similar study observed that improvement in the cognitive-motor function is mediated through SIRT1 activation and increased synaptic activities that also simultaneously showed decreased inflammatory responses (Li et al., 2020).

Evidence from previously published studies offered new opportunities for exploiting EVs as therapeutic and diagnostic tools. This study conducted a systematic review of EVs as diagnostic and therapeutic agents in neurodegenerative diseases.

## Materials and methods

The current systematic review was designed in accordance with the Preferred Reporting Items for Systematic Reviews and Meta-Analyses (PRISMA) recommendations (Page et al., 2021).

### Literature search

By searching PubMed, Scopus, ISI Web of Science, EMBASE, and Google Scholar for all articles on EV modifications, published articles were chosen (2012 to Feb 2022). Figure 1 depicts the number of PubMed publications published in the recent 10 years (2012–2022).

**FIGURE 2:**
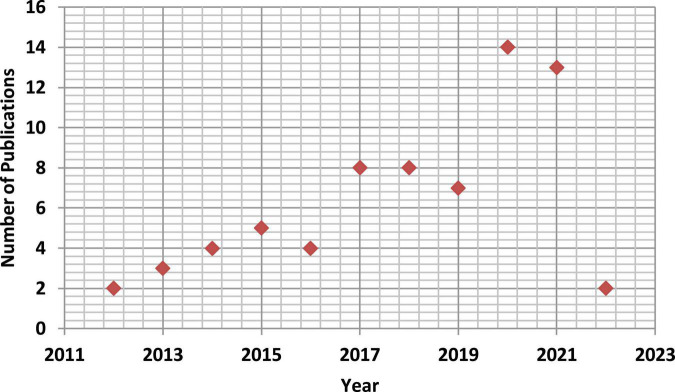
Number of publications for the last 10 years (2012 to 2022) extracted from PubMed that showed the therapeutic and diagnostic role of extracellular vesicles (EVs) in neurodegenerative disease.

Searches were conducted using the following keywords: “extracellular vesicles” [Medical Subject Headings (MeSH)] OR “exosomes” [Medical Subject Headings (MeSH)] OR “Neurodegenerative disease” (MeSH) OR “therapeutics” (MeSH) OR “diagnostic biomarker” (MeSH). Figure 2 demonstrates the number of publications for the last 10 years (2012 to 2022) extracted from PubMed that showed the therapeutic and diagnostic role of EVs in neurodegenerative disease.

Two independent investigators reviewed the titles, aims, and abstracts of published articles to determine eligibility. Full-length articles were evaluated by the same investigators, and inclusion and exclusion criteria were applied to each article. Furthermore, the same researchers screened the references of the initial eligible articles to identify all eligible articles for inclusion in the final list. We calculated the overall risk biasness and inter-reliability agreement.

### Assessment of the risk of bias in the included systematic reviews

The quality of the systematic reviews implicated in the present systematic review was evaluated using the GRADE system (Table 1).

**TABLE 1:** Statistical descriptive analysis showing the assessment of intraclass correlation coefficient, inter-item correlation coefficient, Cohen’s weighted Kappa, and Cronbach’s Alpha assessment between the agreements of the rating experts to analyze reliability statistics.

Assessment	Value	Significance
Intraclass correlation coefficient	0.908 (CI 95%)	< 0.001
Inter-item correlation coefficient	0.931 (CI 95%)	< 0.001
Weighted Kappaa	0.868 (SD.102)	< 0.001
Cronbach’s Alpha	0.906	-

In brief, the GRADE scoring system has four levels of evidence including very low, low, moderate, and high. All eligible studies included in this systematic review were assessed for the following characteristics: imprecision, inconsistency, risk of bias, indirectness, and biasness of publication. Publication bias referred to the application of EVs in the extracted data of reviewed papers and the highlights drawn from the results of such studies because studies without references and publications are not available. The validity of the eligible studies was independently assessed by the two reviewers using a standard checklist. The index of inter-and intra-rater agreement, calculated using the kappa statistic, was used to evaluate the items relevant to the review.

### Inclusion and exclusion criteria

For this study, research papers screened during the literature search met the following inclusion criteria: this study included the following topics: (1) EVs; (2) neurodegenerative diseases; (3) therapeutic and diagnostic biomarkers; (4) exosomes; and (5) a published original article with all full-text literature and properly cited references. The following studies were excluded: (1) insufficient reported data with uncited references; (2) published conference proceedings; (3) published review articles, letters, or text written in a language other than English; or (4) repetition of previously published articles.

### Data extraction

The names of the authors, the year of publication, the country of origin, and the mode of EV application (therapeutic or diagnostic biomarker) were extracted. The majority of articles decipher the role of EVs in neurodegenerative diseases, either as a therapeutic/diagnostic biomarker or both.

## Results

**FIGURE 3:**
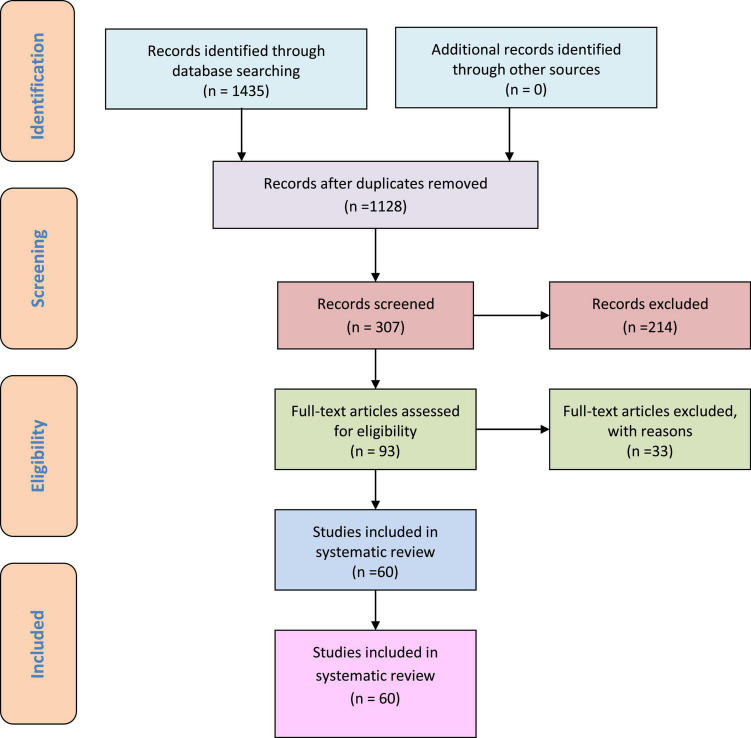
PRISMA flowchart.

A total of 1,435 articles were screened, and 1,128 non-duplicate publications were found. Following the screening of titles and abstracts, 214 publications were excluded; following the full-text screening of 93 published articles, another 33 publications were excluded. As shown in the PRISMA flow diagram, the remaining 60 studies were included in this systematic review (Figure 3).

**FIGURE 4:**
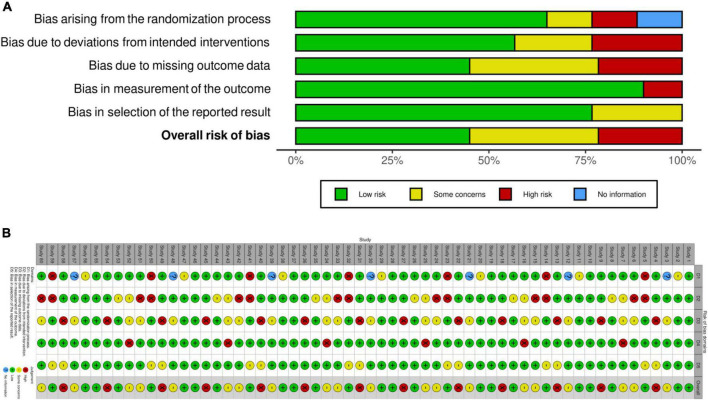
Plot showing risk biasness. **(A)** Weighted bar plots of the distribution of risk-of-bias judgments within each bias domain; **(B)** traffic light plots of the domain-level judgments for each individual result.

Study characteristics of the 27 eligible studies showed that most authors demonstrated the role of EVs as therapeutic and diagnostic biomarkers in neurodegenerative diseases as shown in Table 2 (Candelario and Steindler, 2014; Jan et al., 2017; Lee and Kim, 2017; Izadpanah et al., 2018; Hill, 2019; Jiang et al., 2019; You and Ikezu, 2019; Yuan et al., 2019; Zhang et al., 2019, 2021; Abdel-Haq, 2020; Kapogiannis, 2020; Mäger et al., 2020; Shaimardanova et al., 2020; Vassileff et al., 2020; Yin et al., 2020; Anakor et al., 2021; Cui et al., 2021; D’Anca et al., 2021; Gagliardi et al., 2021; He et al., 2021; Jin et al., 2021; Manu et al., 2021; Toshihide, 2021; Upadhya and Shetty, 2021; Zhao et al., 2021; Picca et al., 2022). In total, 19 studies observed the pioneering role of EVs and exosomes as diagnostic biomarkers in neurodegenerative diseases (Table 2; Coleman and Hill, 2015; Thompson et al., 2016; Vella et al., 2016; Barreca et al., 2017; Chen et al., 2017; Li et al., 2018; Pluta et al., 2018; Ramirez et al., 2018; Gámez-Valero et al., 2019; Karnati et al., 2019; Lee et al., 2019; Pluta and Ułamek-Kozioł, 2019; Pulliam et al., 2019; Vinaiphat and Sze, 2019; Banack et al., 2020; Guedes et al., 2020; Hornung et al., 2020; Rani et al., 2020; Leggio et al., 2021). Moreover, 14 studies alone demonstrated the therapeutic role of EVs and exosomes in neurodegenerative diseases (Table 2; Kim et al., 2013; Katsuda et al., 2015; Rufino-Ramos et al., 2017; Sarko and McKinney, 2017; Araldi et al., 2020; Dolcetti et al., 2020; Lakshmi et al., 2020; Upadhya et al., 2020; Fayazi et al., 2021; Haney et al., 2021; Kumar et al., 2021; Reed and Escayg, 2021; Xu et al., 2021; Meldolesi, 2022). The overall risk biasness was visualized as demonstrated in Figures 4A,B (McGuinness and Higgins, 2021).

**TABLE 2:** Eligible studies included in the systematic review showing the application of extracellular vesicles (EVs) as diagnostic biomarkers and therapeutics or both in neurodegenerative disease.

S. No.	Author, Year, Country	Application of the study (Therapeutics/Diagnostic/Both)	Highlights of the study	References
1	Candelario and Steindler, 2014, USA	Both	Evidence is mounting that EVs can transport mRNAs, miRNAs, non-coding RNAs, and proteins, including those linked to neurodegenerative diseases and cancer, which can then be exchanged between cells. EVs have been found to carry proteins that are prone to aggregation, which is a feature of many neurodegenerative disorders. Patient-specific EVs have the potential to be used as therapeutics in new regenerative medicine protocols for both neurodegenerative diseases.	Candelario and Steindler, 2014
2	Izadpanah et al., 2018, Iran	Both	Because EVs are involved in intercellular communication and a variety of biological processes such as immune response modulation, signal transduction, and transport of low immunogenic genetic materials, they have recently been investigated for the delivery of therapeutic molecules such as siRNAs and drugs in the treatment of diseases. Furthermore, because EV components reflect the physiological state of the cells and tissues that produce them, they can be used as biomarkers for the early detection of various diseases.	Izadpanah et al., 2018
3	Hill, 2019, Australia	Both	Altered genetic cargos, usually in the form of miRNAs, have also been found in EVs patients with these diseases, implying that EVs could be a source of disease biomarkers. Because EVs have been identified as a key pathological contributor to neurological conditions, they will serve as a novel therapeutic target.	Hill, 2019
4	Abdel-Haq, 2020, Italy	Both	Neural-derived blood EVs may be the best strategy for specific, dependable, and early diagnosis of neurodegenerative diseases. EVs have the potential to perform liquid brain biopsy using NDBEVs for early diagnosis and treatment of neurodegenerative diseases.	Abdel-Haq, 2020
5	Shaimardanova et al., 2020, Russia	Both	The contents of neuron-derived extracellular vesicles may indicate pathological changes in the central nervous system, and molecular analysis of extracellular vesicle content aids in the development of non-invasive methods for diagnosing many central nervous system diseases. Extracellular vesicles are a promising vehicle for the delivery of therapeutic substances for the treatment of neurodegenerative diseases and drug delivery to the brain due to their nano size, biosafety, ability to cross the blood-brain barrier, the possibility of targeted delivery, and the lack of an immune response.	Shaimardanova et al., 2020
6	Jin et al., 2021, China	Both	EVs have been shown to transport molecules associated with diseases across the blood-brain barrier (BBB), allowing them to be detected in the blood. Because of this distinguishing feature, they have the potential to serve as diagnostic biomarkers for neurological disorders. EVs derived from mesenchymal stem cells (MSCs) have neurorestorative properties.	Jin et al., 2021
7	Yuan et al., 2019, China	Both	EVs contain proteins linked to the pathogenesis of neurodegenerative diseases (NDs), such as -synuclein (-syn) and tau proteins, implying potential roles for EVs as biomarkers and carriers of drugs and other therapeutic molecules that can cross the blood-brain barrier to treat NDs.	Yuan et al., 2019
8	Upadhya and Shetty, 2021, USA	Both	EVs are also found in cerebrospinal fluid (CSF) and circulating blood, and their identification could lead to the identification of biomarkers linked to specific neurodegenerative diseases. EVs secreted by various stem/progenitor cells contain therapeutic miRNAs and proteins that have shown promise in alleviating symptoms and slowing the progression of neurodegenerative diseases.	Upadhya and Shetty, 2021
9	Vassileff et al., 2020, Australia	Both	EVs and miRNAs have biomarker potential for early diagnosis of these diseases, with stem cell-derived EVs and those generated with exogenous assistance having the most therapeutic potential.	Vassileff et al., 2020
10	You and Ikezu, 2019, USA	Both	EVs have the potential to be used as both a diagnostic marker and a therapeutic agent in neurodegenerative disorders.	You and Ikezu, 2019
11	Manu et al., 2021, Japan	Both	Circulating EVs can be used as a biomarker to monitor MS disease progression and activity, as well as therapeutic reagents or therapy targets.	Manu et al., 2021
12	Zhao et al., 2021, China	Both	Astrocytes-derived EVs contain a high concentration of proteins and nucleic acids that are beneficial to neurons, such as CRYAB and PrP. AEV contents as diagnostic biomarkers for a variety of neurological diseases, identification of key targets for manipulating AEV release, and clarification of various AEV subtypes and their functions	Zhao et al., 2021
13	Anakor et al., 2021, UK	Both	Exosomes are suspected of propagating toxic proteins in neurodegenerative conditions associated with ageing, such as amyotrophic lateral sclerosis (ALS), Alzheimer’s, or Parkinson’s disease. ALS exosomes can be used as a biomarker as well as a therapeutic.	Anakor et al., 2021
14	Lee and Kim, 2017, Republic of Korea	Both	EVs are responsible for the spread of key pathogenic proteins involved in the pathogenesis of amyotrophic lateral sclerosis, Parkinson’s disease, Alzheimer’s disease, and other neurodegenerative disorders. EVs have an advantage over other synthetic drug delivery systems or cell therapy in that they can cross biological barriers such as the blood brain barrier (BBB), modulate inflammation and immune responses, have a longer biodistribution time, and are tumorigenic. EVs have the potential to be used as both a therapeutic and a biomarker in neurodegenerative diseases.	Lee and Kim, 2017
15	Mäger et al., 2020, UK	Both	EVs can be used as diagnostic biomarkers and therapeutics in normal CNS physiology and neurodegenerative diseases, with a focus on Alzheimer’s disease, Parkinson’s disease, multiple sclerosis, amyotrophic lateral sclerosis, and prion diseases.	Mäger et al., 2020
16	Zhang et al., 2021, China	Both	Exosomes play a critical role in CNS cell-cell communication. Exosomes play a dual role in the pathological process of Alzheimer’s disease. Exosomes show promise in the diagnosis and treatment of Alzheimer’s disease.	Zhang et al., 2021
17	Zhang et al., 2019, China	Both	Exosomes are linked to the transmission of disease-related misfolded proteins (such as -synuclein, tau, amyloid -protein, and others) in several neurodegenerative diseases. Exosomes can be used as biomarkers and drug delivery vehicles in the diagnosis and treatment of neurodegenerative diseases.	Zhang et al., 2019
18	Jan et al., 2017, India	Both	EVs have been linked to the transport of various cellular entities across the blood-brain barrier (BBB) and may be useful for delivering drugs and other therapeutic molecules to the brain. EVs also aid in the delivery of disease-causing entities such as prions, -syn, and tau, allowing them to spread to unaffected areas and accelerate the progression of neurodegenerative diseases.	Jan et al., 2017
19	Picca et al., 2022, Italy	Both	The use of EVs for diagnostic and therapeutic purposes may provide unprecedented opportunities for developing personalized approaches.	Picca et al., 2022
20	Jiang et al., 2019, China	Both	EVs shed light on potential treatments for other neurodegenerative diseases. Detection of p-tau and A1–42 can improve diagnostic sensitivity and specificity because these two potential biomarkers are more likely to be delivered to extracellular fluids by exosomes in AD.	Jiang et al., 2019
21	Gagliardi et al., 2021, Italy	Both	Because EVs can enter the systemic circulation and are easily detected in patients’ biological fluids, they have sparked widespread interest as diagnostic and prognostic biomarkers, as well as valuable tools for understanding disease pathogenesis. EVs in amyotrophic lateral sclerosis patients’ blood and cerebrospinal fluid (CSF), implying their potential use in diagnosis and prognosis. EVs may also be used to treat amyotrophic lateral sclerosis.	Gagliardi et al., 2021
22	D’Anca et al., 2021, Italy	Both	EVs contain bioactive molecules (nucleic acids, proteins, and lipids) that affect the recipient cell’s genotype and phenotype. This means that not only EVs themselves, but also their content, may reveal new candidate disease biomarkers and/or therapeutic agents.	D’Anca et al., 2021
23	Toshihide, 2021, Japan	Both	EVs would shed light not only on potential therapeutic targets for neurodegenerative diseases, but also on the development of EV-based biomarkers for disease detection.	Toshihide, 2021
24	Yin et al., 2020, China	Both	Exosomes could aid in the early detection of Alzheimer’s disease and the identification of new therapeutic targets.	Yin et al., 2020
25	Cui et al., 2021, China	Both	Exosomal miRNA content has the potential to be used as a diagnostic and therapeutic tool in neurodegenerative disease.	Cui et al., 2021
26	He et al., 2021, China	Both	Exosomal miRNA content has the potential to be used as a diagnostic and therapeutic tool in neurodegenerative disease.	He et al., 2021
27	Kapogiannis, 2020, USA	Both	EV-based biomarkers are a valuable new tool that will allow researchers to test hypotheses in preclinical proof-of-concept studies with carefully selected participants, propelling therapeutic discovery in neurodegenerative disease.	Kapogiannis, 2020
28	Thompson et al., 2016, UK	Diagnostic	The trafficking of macromolecules from the CNS to the cerebrospinal fluid and blood *via* extracellular vesicles (EVs) represents a promising source of CNS-specific biomarkers, and thus EVs could provide an enriched pool of information about core neuropathogenic, cell-specific processes.	Thompson et al., 2016
29	Vella et al., 2016, Australia	Diagnostic	Exosomes contain proteins associated with neurodegenerative diseases such as prion protein, -amyloid, and -synuclein. Alzheimer’s and Parkinson’s disease, including their possible role in disease propagation and pathology, as well as their utility as a diagnostic in neurodegenerative disease.	Vella et al., 2016
30	Lee et al., 2019, South Korea	Diagnostic	Extracellular vesicles (EVs), which are released by almost all cell types, act as a mediator in the regulation of AD pathogenesis. EV biochemical AD biomarkers, such as proteins and miRNAs. EVs will aid in the early detection of Alzheimer’s disease and the identification of new therapeutic targets.	Lee et al., 2019
31	Vinaiphat and Sze, 2019, Singapore	Diagnostic	EVs and understanding their mechanism of action could pave the way for the discovery of disease-specific biomarkers and therapeutic targets in neurodegenerative disease.	Vinaiphat and Sze, 2019
32	Pulliam et al., 2019, USA	Diagnostic	EVs derived from neural cells have the potential to be exciting biomarkers of neurodegeneration in Alzheimer’s disease.	Pulliam et al., 2019
33	Ramirez et al., 2018, USA	Diagnostic	EVs released from all circulating blood elements, as well as the luminal surface of the endothelium, can be used as biomarkers in CSF.	Ramirez et al., 2018
34	Banack et al., 2020, USA	Diagnostics	Neural-enriched extracellular vesicles may provide microRNA (miRNA) fingerprints with unequivocal neurodegeneration signs that can be used as a biomarker.	Banack et al., 2020
35	Barreca et al., 2017, Italy	Diagnostics	Because EVs are involved in the pathogenesis of multiple sclerosis, they could be used as a biomarker.	Barreca et al., 2017
36	Guedes et al., 2020, USA	Diagnostic	Exosomes, which can cross the blood-brain barrier and be isolated from peripheral fluids such as serum, saliva, sweat, and urine, are promising TBI biomarkers.	Guedes et al., 2020
37	Gámez-Valero et al., 2019, Spain	Diagnostic	Specific biomarkers will aid in the resolution of overlapping features of various dementias. Extracellular vesicles (EVs) are a dependable and stable biomarker source that can be found in a variety of bodily fluids. EV proteins and miRNAs may define the pathology’s specific biosignature.	Gámez-Valero et al., 2019
38	Karnati et al., 2019, Maryland	Diagnostic	EVs enriched for neuronal origin can be extracted from peripheral blood samples and their contents quantified as a window into potential brain changes. Exosomal proteins and microRNAs (miRNAs) may be novel biomarkers to aid in the clinical diagnosis and treatment response of neurological disorders.	Karnati et al., 2019
39	Pluta et al., 2018, Poland	Diagnostic	Exosomes secreted in Alzheimer’s disease may aid in spreading and progression, highlighting their potential utility as future diagnostic antemortem biomarkers in this devastating disease.	Pluta et al., 2018
40	Hornung et al., 2020, USA	Diagnostic	CNS-derived exosomes have also been shown to cross the blood-brain barrier into the bloodstream, attracting significant interest as a source of biomarkers for various neurodegenerative diseases because they can be isolated using a minimally invasive blood draw.	Hornung et al., 2020
41	Li et al., 2018, China	Diagnostic	Amyloid -protein, -synuclein, Huntington-associated protein 1, and superoxide dismutase I can be transported to other cells by exosomes in the CNS. The network of exosomes that regulates CNS homeostasis is a promising biomarker for neurodegenerative disease diagnosis and treatment.	Li et al., 2018
42	Coleman and Hill, 2015, Australia	Diagnostic	Exosomes also play a role in the processing of the amyloid precursor protein (APP), which is linked to Alzheimer’s disease (AD). Exosomes are a potential source of biomarkers for neurological conditions because they can be isolated from circulating fluids such as serum, urine, and cerebrospinal fluid (CSF).	Coleman and Hill, 2015
43	Leggio et al., 2021, Italy	Diagnostic	Parkinson’s disease patient-derived EVs derived from a variety of biological specimens can be investigated as diagnostic biomarkers.	Leggio et al., 2021
44	Pluta and Ułamek-Kozioł, 2019, Poland	Diagnostic	EVs can be used as biomarker in AD.	Pluta and Ułamek-Kozioł, 2019
45	Rani et al., 2020, India	Diagnostic	Exosomes can be utilized in the diagnosis of neurodegenerative disorders due to their easy availability from most biological fluids such as blood, urine, saliva, breast milk, sperm, and so on, their extremely high disease-specific bio-molecular signature/profile, exosomes’ ability to cargo a variety of biomolecules in between cells, and their ability to cross the blood-brain barrier.	Rani et al., 2020
46	Chen et al., 2017, China	Diagnostic	Exosomal miRNAs in Alzheimer’s disease pathology exploits the potential of these miRNAs as diagnostic biomarkers in Alzheimer’s disease, as well as the use of exosomes in miRNA delivery, which may lead to significant advances in the field of macromolecular drug delivery.	Chen et al., 2017
47	Rufino-Ramos et al., 2017, Portugal	Therapeutics	EVs linked to AAV vectors (vexosomes), enveloped protein nanocages (EPNs), and exosome-mimetic nanovesicles can be used as therapeutic vehicles.	Rufino-Ramos et al., 2017
48	Lakshmi et al., 2020, India	Therapeutics	The ability of exosomes to increase A clearance suggests a novel therapeutic role for exosomes in Alzheimer’s disease intervention.	Lakshmi et al., 2020
49	Kumar et al., 2021, USA	Therapeutics	Because of their biocompatibility, stability, and targeted delivery with limited immunogenicity, as well as their ability to be delivered *via* a non-invasive approach for the treatment of neurodegenerative diseases, EVs can be used as a drug delivery system.	Kumar et al., 2021
50	Dolcetti et al., 2020, Italy	Therapeutics	EVs as potential therapeutic targets and tools for therapeutic intervention in multiple sclerosis.	Dolcetti et al., 2020
51	Upadhya et al., 2020, USA	Therapeutics	Naive astrocytes release EVs containing a variety of neuroprotective compounds such as fibroblast growth factor-2, vascular endothelial growth factor, and apolipoprotein-D. When astrocytes are stimulated, they secrete EVs containing neuroprotective molecules such as heat shock proteins, synapsin 1, unique microRNAs, and glutamate transporters. Astrocyte-derived EVs (ADEVs) derived from specific culture conditions, as well as ADEVs engineered to carry desired miRNAs or proteins, are likely to be useful in treating brain injury and neurodegenerative disease.	Upadhya et al., 2020
52	Sarko and McKinney, 2017, USA	Therapeutics	Exosomal content has been shown to aid in the promotion of neurodegeneration pathways such as -amyloid peptide (A) accumulation forming amyloid plaques in Alzheimer’s disease brains and pathological aggregates of proteins containing -synuclein in Parkinson’s disease transferred to the central nervous system *via* exosomes. Exosomes can cross the blood-brain barrier, can be strategically engineered to carry drugs or other treatments, and have an appropriate half-life and stability for this purpose.	Sarko and McKinney, 2017
53	Araldi et al., 2020, Brazil	Therapeutics	Stem cell-derived exosomes can be used as therapeutic approach for Neurodegenerative Disorders.	Araldi et al., 2020
54	Xu et al., 2021, China	Therapeutics	Bioengineered EVs can have the ability to cross BBB and can be effectively used for therapeutic purposes.	Xu et al., 2021
55	Meldolesi, 2022, Italy	Therapeutics	Stem cells contains blockers of the enzyme BACE-1, that induce, in neurons and glial cells and decreased levels of Aβ, the key peptide of the Alzheimer’s disease and a resultant showed therapeutic effect.	Meldolesi, 2022
56	Fayazi et al., 2021, Iran	Therapeutics	Stem cell-derived exosomes can be explored in treatment of various neurodegenerative diseases.	Fayazi et al., 2021
57	Haney et al., 2021, USA	Therapeutics	EVs secreted from macrophages are suggested as the most promising nanocarrier system for drug delivery to the brain	Haney et al., 2021
58	Reed and Escayg, 2021, Georgia	Therapeutics	EVs attenuate reactive gliosis, neuronal death, pro-inflammatory signaling, as well as reduce cognitive, behavioral, and motor deficits. EVs can be used as therapeutics in neurodegenerative diseases	Reed and Escayg, 2021
59	Kim et al., 2013, South Korea	Therapeutics	MSCs secrete trophic factors and cytokines (secretome) that have therapeutic relevance for the neurogenic, neuroprotective, angiogenic and anti-inflammatory/immunoregulatory activities. MSCs secreted exosomes have therapeutic role in neurodegenerative disease.	Kim et al., 2013
60	Katsuda et al., 2015, Japan	Therapeutics	EVs secreted from human adipose tissue-derived MSCs (hADSCs) (also known as adipose tissue-derived stem cells; ASCs) against Alzheimer’s disease (AD). hADSCs secrete exosomes carrying enzymatically active neprilysin, the most important β-amyloid peptide (Aβ)-degrading enzyme in the brain	Katsuda et al., 2015

The systematic review of the published articles was found consistent in validity appraisal among the two raters, as assessed using a kappa statistic of 0.868. Inter-reliability coefficient and intra-reliability coefficient showed strong agreement between the reviewer’s decisions as shown in Table 1. Table 1 also shows that Cronbach’s alpha (α) coefficient measures reliability and internal consistency and demonstrates excellent reliability levels.

## Discussion

In the present systematic review, the role of EVs in neurodegenerative diseases was evaluated. Altogether, the findings suggested that EVs can be used both as diagnostic biomarkers and therapeutics in neurodegenerative diseases. EVs are nano-sized lipid bilayer vesicles sized between 30 and 1,000 nm that are released by several cell types including healthy and diseased cells. These secreted EVs play an important role in intercellular communication mediated through their biological content (protein, nucleic acids, and lipids). Previous ample evidence suggested that EVs are the carrier of mRNAs, miRNAs, long non-coding RNAs, and proteins that are associated with the neurodegenerative changes in the body, which may be exchanged between cells of the target. Bestowing this reason, EVs attract special space for acting both as diagnostic markers and therapeutics in neurodegenerative diseases. The majority of studies observed the role of EVs in both diagnostics and therapeutics (Candelario and Steindler, 2014; Jan et al., 2017; Lee and Kim, 2017; Izadpanah et al., 2018; Hill, 2019; Jiang et al., 2019; You and Ikezu, 2019; Yuan et al., 2019; Zhang et al., 2019, 2021; Abdel-Haq, 2020; Kapogiannis, 2020; Mäger et al., 2020; Shaimardanova et al., 2020; Vassileff et al., 2020; Yin et al., 2020; Anakor et al., 2021; Cui et al., 2021; D’Anca et al., 2021; Gagliardi et al., 2021; He et al., 2021; Jin et al., 2021; Manu et al., 2021; Toshihide, 2021; Upadhya and Shetty, 2021; Zhao et al., 2021; Picca et al., 2022). Notably, 19 studies observed the pioneering role of EVs as a diagnostic biomarker in neurodegenerative diseases (Table 2; Coleman and Hill, 2015; Thompson et al., 2016; Vella et al., 2016; Barreca et al., 2017; Chen et al., 2017; Li et al., 2018; Pluta et al., 2018; Ramirez et al., 2018; Gámez-Valero et al., 2019; Karnati et al., 2019; Lee et al., 2019; Pluta and Ułamek-Kozioł, 2019; Pulliam et al., 2019; Vinaiphat and Sze, 2019; Banack et al., 2020; Guedes et al., 2020; Hornung et al., 2020; Rani et al., 2020; Leggio et al., 2021). Moreover, 14 studies alone demonstrated the therapeutic role of EVs in neurodegenerative diseases (Table 2; Kim et al., 2013; Katsuda et al., 2015; Rufino-Ramos et al., 2017; Sarko and McKinney, 2017; Araldi et al., 2020; Dolcetti et al., 2020; Lakshmi et al., 2020; Upadhya et al., 2020; Fayazi et al., 2021; Haney et al., 2021; Kumar et al., 2021; Reed and Escayg, 2021; Xu et al., 2021; Meldolesi, 2022).

One of the previously published studies performed on cell culture showed the presence of proteins susceptible to aggregation, which was proven a classical hallmark in neurodegenerative diseases (Funk and Kuret, 2012). A diseased or affected cell transport protein to a healthy cell may further lead to its accumulation and aggregation, thereby causing the pathogenesis of neurodegenerative diseases (Funk and Kuret, 2012). Cerebrospinal fluid (CSF) and blood specimens from patients often implicate the screening and identification of biomarkers. Due to some limitations of other interacting factors and non-specificity, these might not be suitable candidates for the diagnosis of neurodegenerative disease as EVs do. Similarly, the low concentration of nucleic acids in such fluids also limits the use of such biomarkers in the screening of neurodegenerative changes.

Nonetheless, EVs are considered to be an ideal reservoir for specific biomarkers of neurodegenerative changes. EVs are released by all cells including the CSF, blood, and urine, which will be a beneficial approach for the detection of the brain and neural cell-related changes. EVs are resistant to enzymatic degradation and thereby provide stability during the processing and isolation of EV biomarkers. Therefore, EVs explore the potential of liquid biopsy for the investigation of neurodegenerative diseases.

A previously published study observed that EVs of neurodegenerative diseases exhibit the presence of pathogenic proteins including α-synuclein (α-syn) and tau proteins, which serve as diagnostic biomarkers and therapeutic targets in such diseases (Yuan et al., 2019). Over the past few decades, it was observed that neuronal cell-derived EVs possess Aβ42, T-tau, and P-T181-tau exploited as diagnostic biomarkers.

Extracellular vesicles possess the ability to cross the blood-brain barrier (BBB) and have the potential to efficiently deliver therapeutic molecules into the cells providing an attractive opportunity for delivering mRNAs, miRNAs, and drugs to show a protective effect in neurodegenerative diseases. A previously published study showed that astrocyte-derived EVs (AEVs) exhibit neuroprotection in ischemic stroke conditions (Zhao et al., 2021). AEVs showed neuroprotection in ischemic stroke conditions by controlling autophagy, the release of miR-92b-3p, assuaging oxygen-glucose deprivation-induced neuron apoptosis, and inhibiting the expression of TNFα, IL-6, and IL-1β that will further lead to the reduction of infarct volumes (Zhao et al., 2021). Exploring the new area of EV biology seems crucial for scientific and break-throwing discoveries in a variety of diseases including neurodegenerative diseases. EVs from every cellular origin should be exploited for diagnostic and therapeutic in neurodegenerative diseases.

Extracellular vesicles are known to play a dual role in diagnostics as well as therapeutics in neurodegenerative diseases. In one of the previously published studies, it was demonstrated that intraperitoneal injection of EVs derived from umbilical cord MSCs showed improved cognitive outcomes through the decrease in neurological severity scores and improved reflex and sensation mediated by an HDAC1-Dependent EGR2/Bcl2 Axis (Han et al., 2021). Similarly in the rodent model, treatment with EVs showed a decrease in 180° rotation time compared to untreated animals in behavioral negative geotaxis tests (Sisa et al., 2019). Xin and his coworkers demonstrated that EVs derived from MSCs attenuate hindlimb impairment along with improvement in perception and visual impairment by modulating microglia/macrophage polarization and targeting the delivery of miR-21a-5p (Xin et al., 2020).

## Conclusion

Extracellular vesicles exhibit promising therapeutic and diagnostic uses in biomaterials. These tailored nano-vesicles can be loaded with desired biomolecules, such as proteins, lipids, nucleic acids, and drugs, using different modification approaches to obtain functionalized EVs. These functionalized EVs can be exploited in the treatment of various diseases, including neurodegenerative diseases. In the recent decade, there has been an increasing trend of research into exosomes, which has expanded into neurodegenerative diseases. The body of evidence demonstrates that exosomes play an important part in communication in the brain. On understanding the physical nature of exosomes, it may be possible to manipulate their contents to deliver therapeutic factors to delay the onset of neurodegeneration. Bringing the field of immunology, neurology, and oncology along with infectious diseases could help in exploiting all the aspects of EVs for giving an understanding pathogenesis and management of human diseases. Combining immunology, neurology, oncology, and infectious diseases could aid in the exploitation of all aspects of EVs for understanding the pathogenesis and management of human diseases.

## Data availability statement

The original contributions presented in this study are included in the article/supplementary material, further inquiries can be directed to the corresponding author.

## Author contributions

AR, G-BJ, MS, RG, and SA: conceptualization. AR and G-BJ: methodology. AR: software, investigation, and writing—original draft preparation. G-BJ, KG, SA, MS, RG, and SK: validation. G-BJ, SA, MS, RG, and SK: formal analysis. SK: resources and project administration. AR and KG: data curation. KG, RG, SA, MS, and G-BJ: writing—review and editing. SA, MS, RG, and SK: visualization. SA, MS, RG, G-BJ, and SK: supervision. All authors contributed to the article and approved the submitted version.

## Abbreviations

SF: synovial fluid
BAL: bronchoalveolar lavage
AF: amniotic fluid
PE: pleural effusions
EVs: extracellular vesicles
CNS: central nervous system
ILVs: intraluminal vesicles
MVBs: multivesicular bodies
ESCRT: endosomal sorting complex required for transport
AD: Alzheimer’s disease
PRISMA: preferred reporting items for systematic reviews and meta-analysis
CSF: cerebrospinal fluid
BBB: blood brain barrier
AEVs: astrocyte EVs.
